# Radiographic and Functional Outcomes Following Resorbable Screw-Augmented Suture Fixation vs. All-Suture Fixation of Mid-pole Patellar Fractures: A Comparative Case Series

**DOI:** 10.7759/cureus.70956

**Published:** 2024-10-06

**Authors:** Daran Huang, Hun Yi Koh, Bing Howe Lee, Hamid Rahmatullah Bin Abd Razak

**Affiliations:** 1 Orthopaedic Surgery, Sengkang General Hospital, Singapore, SGP; 2 Musculoskeletal Sciences, Duke-Nus Medical School, Singapore, SGP

**Keywords:** complications, implants, orthopaedic trauma, outcomes, patellar fracture, suture fixation, tension band, union

## Abstract

Purpose

Midpole patellar fractures are traditionally fixed with an “11-8” metal tension band construct. However, this technique is rife with implant-related complications. This study aims to evaluate the radiographic and functional outcomes following “all-suture” fixation of mid-pole patellar fractures as compared to resorbable screw-augmented suture fixation.

Methods

We retrospectively studied a consecutive series of 18 patients, 9 each with mid-pole patellar fractures treated with all-suture fixation or suture fixation augmented with bioabsorbable cancellous screws in our institution. The hybrid fixation cohort was significantly older (p<0.01). Radiographic and functional outcomes, such as time to union, postoperative range of motion (ROM), and the presence of complications such as fracture displacement were recorded and evaluated. The minimum follow-up was one year.

Results

All cases achieved radiographic union by 15 weeks postoperatively except one from the hybrid fixation cohort. The average time to radiographic union was comparable (p=0.30). Twenty-two point two percent (22.2%; 2 out of 9) of the cases from each cohort had an increase in the fracture gap (>2 mm) at around four to six weeks postoperatively, for which all except one case from the hybrid fixation cohort achieved union thereafter. One patient from the hybrid fixation cohort had fibrous non-union and further fracture displacement. There was another case of mild fracture gapping and screw breakage on review of postoperative radiographs at three months from the hybrid fixation cohort. These patients recovered without surgical revision or implant removal.

Conclusions

Both non-metal fixation methods for mid-pole transverse patellar fractures proved to be radiographically and functionally comparable.

## Introduction

The patella is a large sesamoid bone that plays a crucial role in knee extension. Mid-pole patellar fractures are a common injury that can lead to debilitating impairment [1-3]. These fractures are mostly a result of direct forceful trauma such as a fall when the knee is bent. For non-displaced fractures, whereby the extension mechanism of the knee is preserved, conservative treatments like immobilization with a knee brace or cast may be sufficient [4-6]. Most cases would, however, require surgical intervention, traditionally via metallic implants, such as Kirschner (K-wire) or cerclage wire fixation, in a tension band construct [2,7,8]. This method has been revolutionized by Arbeitsgemeinschaft für Osteosynthesefragen (AO), mainly involving the application of two longitudinal inter-fragment K-wires and an anterior figure-of-eight cerclage wire loop to act as the tension band in a “11-8” manner [9]. Although such a construct yields a satisfactory union rate of up to 87.5%, it is plagued by several complications [10]. These include incidences of implant breakage and migration leading to prominence over time, which often necessitates a second surgery for revision or implant removal, compounding the healthcare burden [11]. Therefore, surgeons have begun to seek non-metallic alternatives such as sutures to mitigate these complications.

In our institution, an all-suture fixation technique has been implemented for mid-pole patellar fractures and concluded to be a viable alternative with satisfactory union in addition to not requiring revision surgeries [12]. An alternative would be the use of bioabsorbable cannulated lag screw fixation. Qi et al. and Usami et al. reported their series on the use of a combination of bioabsorbable cannulated lag screws and nonabsorbable suture tension bands, which also resulted in comparable outcomes [13,14]. However, they did not include a comparison group. Our study aims to prospectively compare and evaluate radiographic and functional outcomes between the screw-augmented suture (hybrid) fixation versus all-suture fixation techniques. We hypothesize that there are no differences between all-suture and hybrid fixation in terms of postoperative functional scores, union rates, and complication rates.

## Materials and methods

Ethics approval and subjects

The SingHealth Centralised Institutional Review Board approved the conduct of this study (2022/2214). This study examined all patients admitted to our institution from July 2018 to January 2020 with mid-pole patellar fractures who were treated with all-suture fixation or suture fixation augmented with bioabsorbable cancellous screws. Clinical data on radiographic and functional outcomes, such as time to union and postoperative range of motion (ROM), and the presence of complications such as fracture displacement were recorded using the national electronic health record system and evaluated.

Surgical technique

**Figure 1:**
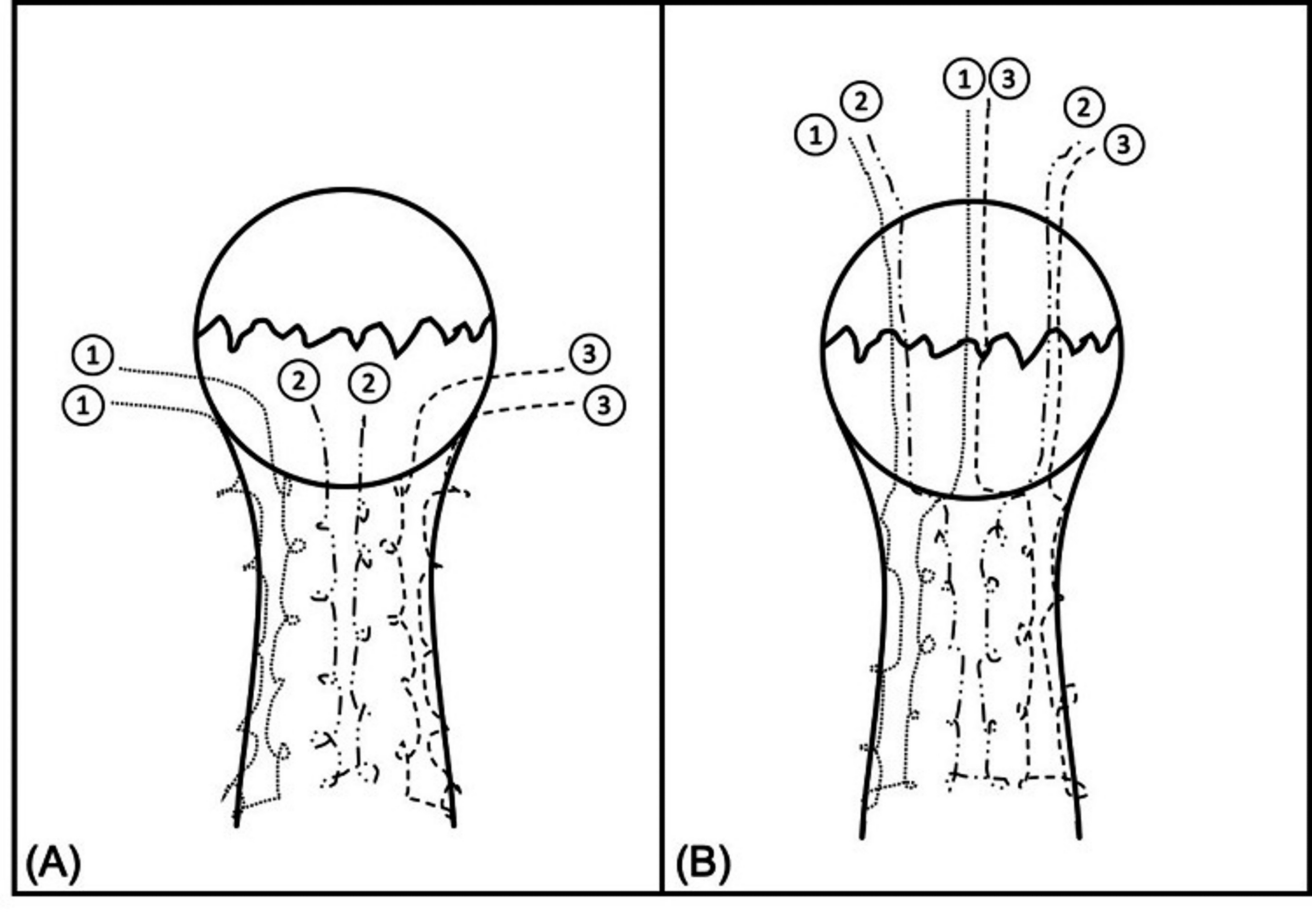
Illustration of the Krackow locking stitch suture technique (A) The patellar tendon is reinforced with six strands of non-absorbable, high-tensile strength sutures, configured in a locking stitch. (B) After being threaded through the bone, the cross-matched sutures are repositioned to the superior pole of the patella. Source: [12] (under CC-BY 4.0 license)

All patients, regardless of intervention cohort, were treated with consistent principles and techniques of fracture reduction and fixation. Surgeries were performed by three fellowship-trained orthopedic surgeons. Patients were positioned supine on a radiolucent table with a pneumatic tourniquet applied to the proximal thigh of the affected limb. Dissection down to the fracture site was made through a longitudinal midline incision to the skin. The joint and fracture site was copiously irrigated of hematoma and debris. The fractured patellar fragments were reduced in situ with a pointed bone reduction clamp. In cases where there were no existing tears in the patellar retinaculum, a separate incision was made on the retinaculum to assess for articular congruity post-reduction. Realignment was confirmed to be satisfactory via fluoroscopy. Strong non-absorbable sutures like Ultrabraid^®^ (Smith & Nephew, Memphis, TN, USA), FibreWire^®^ (Arthrex, Naples, FL, USA), and/or No. 5 Ethibond® (Ethicon, Somerville, NJ, USA) were utilized to stitch the patella tendon from the distal pole of the patella to the tibial tuberosity to fashion a Krackow locking suture, as seen in Figure 1.

These sutures were subsequently cross-matched and passed transosseously through the fracture site using a 2.7mm beath pin, creating three parallel longitudinal suture tunnels. Afterward, the sutures were separated and tied "like-to-like" superior to the patella, with the knots then buried within the quadriceps tendon.

**Figure 2:**
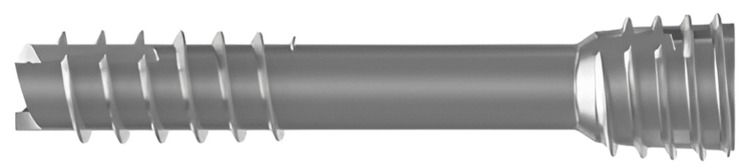
Illustration of the Magnezix® cerclage suture This screw is constructed from a fully bioabsorbable magnesium-based alloy, also comprised yttrium, rare earth elements, and zirconium (MgYREZr).

For the bioabsorbable screw augmented suture group, two guide wires were inserted longitudinally across the fracture site in a parallel fashion after fracture reduction. Two cannulated headless compression screws (HCS, Magnezix, Syntellix AG, Hanover, Germany), as seen in Figure 2, were then implanted across the guidewires. High-strength non-absorbable sutures were likewise employed to stitch the patella tendon from the distal pole of the patella to the tibial tuberosity using a Krackow locking suture. These sutures were cross-matched and passed transosseously through the cannulated screws using a 2.7mm beath pin, creating three parallel longitudinal suture tunnels within the screws. Subsequently, the sutures were untangled and tied similarly to the all-suture technique. In addition, an extra non-absorbable suture was employed for additional stability as a "figure-of-eight" cerclage suture in certain instances. Fluoroscopy was again deployed to confirm articular restoration before wound closure.

Postoperative protocol

Patients were prescribed a ranging knee brace postoperatively and permitted to weight bear as tolerated in it from the first postoperative day (POD1). They were counseled to refrain from active quadriceps contraction for the first two weeks. They were discharged and then followed up fortnightly with serial radiographs to monitor fracture alignment and healing. The increment in ROM was calibrated to the radiographic progression and the fracture pattern (i.e., ROM 0-30° for two weeks; 0-60° from weeks 3-4; 0-90° from weeks 4-6; beyond 90° after six weeks titrated to patient and fracture factors).

Radiographic assessment

**Figure 3:**
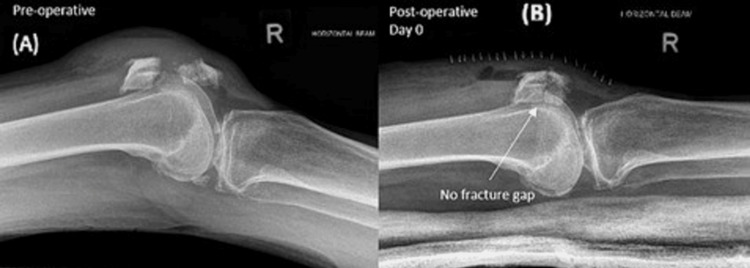
Radiograph of a mid-pole transverse patellar fracture fixed with the all-suture technique (A) Preoperative X-ray showing the mid-pole displaced transverse patellar fracture (B) Postoperative X-ray after suture fixation of the patellar fracture

Orthogonal radiographs were taken fortnightly from POD1 until 12 weeks postoperatively, or when a radiographic union was achieved, defined as the presence of cortical and trabecular bridging across the fracture site, callus formation, and fading of the fracture line [15]. The degree of fracture displacement was quantified on lateral radiographs; an articular displacement of 2mm or more was deemed significant. The union rate was tabulated by deriving the proportion of patients with complete healing at 12 weeks postoperatively. Figure 3 shows a successful case.

Functional assessment

Validated patient-reported outcome measures (PROMs) including the EuroQol-5 Dimensions (EQ-5D) and Oxford Knee Score (OKS) were collected [16,17]. The authors are from a public institution with a specialized Orthopaedic Diagnostic Centre that was set up with a license to use the OKS. These scores were tabulated by a co-author (KHY) based on postoperative recall (by patient or caregiver after their latest follow-up) following a standardized questionnaire script.

Statistical analysis

Data analysis was performed using IBM SPSS Statistics version 22 (IBM Corp., Armonk, NY, US). Unpaired, parametric sample T-tests were conducted on the datasets to detect significant differences between both intervention cohorts for scores of EQ-5D and OKS, time to union (weeks), and ROM (degrees). All reported p-values were two-tailed, and a significance level of <0.05 was considered significant.

## Results

A total of 18 consecutive cases (9 all-suture fixations vs. 9 hybrid fixations) were available for review, with a minimum follow-up of one year. There were nine males and females each.

On average, the hybrid fixation cohort was significantly older (70.8 ± 10.4 years) than the all-suture fixation cohort (54.1 ± 9.6 years; p<0.01, t=3.5). The mean ROM of the knee was 0 to 96.7 (± 10.0) and 0 to 104 (± 12.7) degrees respectively at one year (p=0.10). All cases achieved radiographic union by 15 weeks postoperatively except one from the hybrid fixation cohort (100% vs 88.9%). The average time to radiographic union was 6.4 ± 1.7 and 7.1 ± 3.2 weeks respectively (p=0.30). 22.2% (2 out of 9) of the cases from each cohort had an increase in the fracture gap (>2 mm) at around 4 to 6 weeks postoperatively. Two of the all-suture fixation cases had an eventual union at 8 and 10 weeks postoperatively while one case from the hybrid fixation cohort achieved union at 6 weeks. The last patient from the hybrid fixation cohort had fibrous non-union and further displacement of the fracture fragments, presumably as he was not compliant with the ROM restriction of the postoperative protocol. Fortunately, this patient was last reviewed one year postoperatively and had clinical union without any functional limitations.

**Figure 4:**
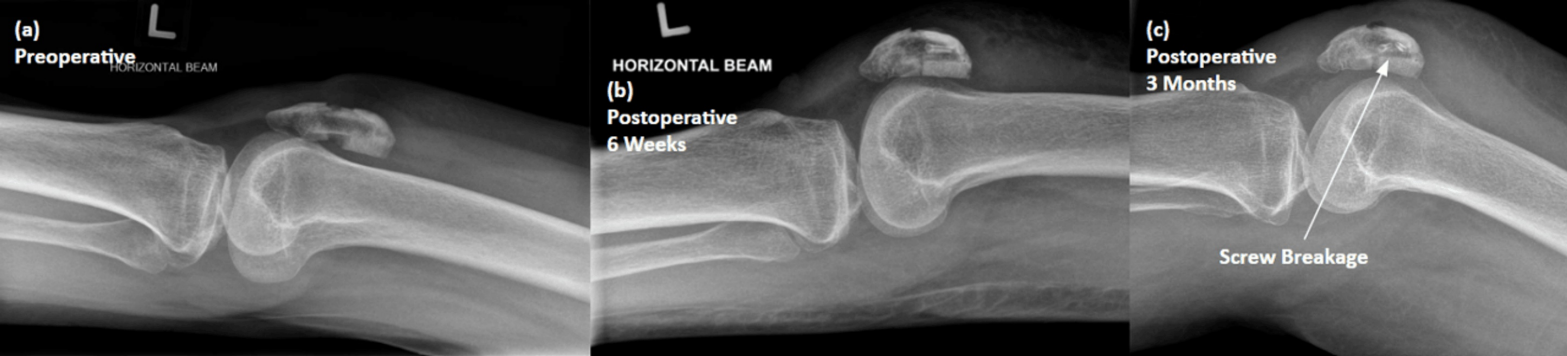
Radiograph of a mid-pole transverse patellar fracture fixed with a resorbable screw-augmented suture technique (A) Preoperative X-ray showing the mid-pole displaced transverse patellar fracture (B) Six weeks postoperative X-ray after hybrid fixation of the patellar fracture (C) Three months postoperative X-ray showing breakage of the screw, new since the previous radiograph, though there is interval healing of the fracture

One patient from the hybrid fixation cohort had mild fracture gapping as well as screw breakage on review of postoperative radiographs at 3 months (Figure 4). She recovered unremarkably without the need for any surgical revision or implant removal.

One year postoperatively, none of the patients reported issues with suture prominence, and there was no need for any revision surgery. A summary of the clinical data of the 2 cohorts of the 18 patients included in this study is presented in Tables 1, 2.

**Table 1:** Clinical data of the all-suture fixation cohort EQ-5D: EuroQol-5 Dimensions; OKS: Oxford Knee Score

S. No (all-suture)	Age (years)	Gender	Side	Fracture union	Time to union (weeks)	Fracture displacement	Postop complications	Revision surgery	EQ-5D	OKS
1	43	Male	Left	Yes	4	No	No	No	-	-
2	61	Female	Right	Yes	8	Yes	No	No	1	47
3	60	Female	Right	Yes	6	No	No	No	0.847	37
4	38	Male	Left	Yes	6	No	No	No	1	48
5	54	Male	Right	Yes	6	No	No	No	1	48
6	61	Female	Left	Yes	10	Yes	No	No	-	-
7	63	Female	Left	Yes	6	No	No	No	1	48
8	45	Male	Left	Yes	6	No	No	No	0.809	43
9	62	Male	Right	Yes	6	No	No	No	1	46

**Table 2:** Clinical data of the resorbable screw augmented suture fixation cohort EQ-5D: EuroQol-5 Dimensions; OKS: Oxford Knee Score

S. No. (hybrid)	Age (years)	Gender	Side	Fracture union	Time to union (weeks)	Fracture displacement	Postop complications	Revision surgery	EQ-5D	OKS
1	84	Male	Right	No	No	Yes	No	No	-	-
2	54	Female	Left	Yes	6	Yes	Mild fracture gapping and screw breakage	No	0.5	26
3	60	Female	Left	Yes	6	No	No	No	0.938	45
4	72	Male	Left	Yes	15	No	No	No	-	-
5	64	Female	Left	Yes	6	No	No	No	1	44
6	85	Female	Right	Yes	6	No	No	No	1	41
7	68	Male	Right	Yes	6	No	No	No	-	-
8	74	Female	Right	Yes	6	No	No	No	0.847	40
9	76	Male	Right	Yes	6	No	No	No	-	-

As for functional outcomes, the mean EQ-5D was 0.951 ± 0.085 for all-suture fixation vs 0.857 ± 0.209 for hybrid fixation at 1 year postoperatively (p=0.19). The mean OKS was 45.3 ± 4.1 vs 39.2 ± 7.7, respectively (p=0.08). There were no statistical differences for EQ-5D and OKS between both groups.

A summary of the statistical comparison between the all-suture and hybrid fixation cohorts is presented in Table 3.

**Table 3:** Statistical comparison between the all-suture fixation and resorbable screw-augmented suture fixation cohorts Bolded asterisked value(s)* indicate statistically significant results.

	All-suture fixation (n = 9)	Hybrid fixation (n = 9)	p-value	t-value
Age (years)	54.1 ± 9.6	70.8 ± 10.4	<0.01*	3.5*
Gender (M:F)	4 : 5	5 : 4		
Time to union (weeks)	6.4 ± 1.7	7.1 ± 3.2	0.30	0.6
ROM (degrees)	96.7 ± 10.0	104 ± 12.7	0.10	1.3
EQ-5D	0.951 ± 0.085	0.857 ± 0.209	0.19	1.2
OKS	45.3 ± 4.1	39.2 ± 7.7	0.08	2.1

## Discussion

This study documented that the “all-suture” and resorbable screw-augmented suture fixation techniques were comparable in terms of outcomes and are practical alternatives to the treatment of mid-pole patella fractures.

We had previously reported excellent radiographic union rates and no complication rates in our series of all-suture fixation [12]. An augmentation to all-suture fixation is the use of bioabsorbable screw fixation, for which various technical aspects warrant consideration. They are known for their superior fracture reduction, attributed to the fact that the forces generated by the cannulated screws act perpendicularly to the fracture line, resulting in consistent forces on the articular surface of the patella. There is also improved stability and reduced likelihood of fracture dislocation from the lag screws than using only sutures, allowing for earlier knee joint rehabilitation. Importantly, both techniques reduce the incidence of complications that easily arise from the usage of metal implants and negate the need to remove the implants [13]. Usami et al. also suggested that bioabsorbable screw fixation is clinically safe with no instances of late aseptic swelling, foreign body reactions, or tissue reactions to the implants [14]. In our institution, we have begun implementing this bioabsorbable screw augmented suture (hybrid) fixation technique for mid-pole patellar fractures.

However, there can be issues with the use of bioabsorbable screws as well. Correctly positioning these screws can be technically challenging, especially when fracture fragments are not sufficiently sizable to hold multiple screws [18]. This is complicated by the consensus that the patella tends to be smaller in our Asian population, limiting surgeons to only the smallest screw sizes for good fitting [19,20]. Another potential issue is the ability of the biodegradable implant to hold its structural integrity to provide sufficient stability for fracture healing; cases of screw migration and even peri-implant fracture have been documented [18]. Nevertheless, while the anticipated time frame for full degradation is approximately one year as indicated in an animal study, subsequent MRI scans conducted at the 36-month mark demonstrated that the former implant sites have become filled with bone tissue comprising both partially cancellous and cortical elements despite degradation of the implants [21]. Altogether, biodegradable implants can be a safe and reliable option for fracture fixation.

To our best knowledge, no literature has directly compared all-suture versus hybrid fixation with screw augmentation. In this study, a radiographic union was observed in all but one case from the hybrid fixation cohort (100% vs 88.9%) by 15 weeks, with no statistically significant difference in average time to radiographic union. These findings align well with union rates and fixation times reported in the present literature. In a prospective study by Li et al. on hybrid fixation in 15 patients via bioabsorbable cannulated lag screws and braided polyester suture tension bands, radiographic union occurred in all cases approximately 3 months postoperatively, with no instances of implant failure [13]. In the study by Chen et al., which included 25 patients who underwent transosseous suturing for displaced patellar fractures, there were similar union times of 8.4 ± 2.9 weeks [22]. These results suggest that in terms of union time, there is non-inferiority in either fixation method for patellar fractures.

Two patients from each intervention cohort experienced an increase in the fracture gap, identified between four to six weeks postoperatively. Among these cases, three eventually achieved union, with only one exhibiting fibrous non-union radiographically at the 12-week mark. The occurrence of fracture gapping in suture fixation had been previously documented by Camarda et al., who observed a reduction loss (<4 mm) at four weeks postoperatively before eventual union [23]. This phenomenon may be attributed to potential soft tissue interposition of the sutures, resulting in loosening following soft tissue atrophy [24]. Nonetheless, this fracture displacement proved to be transient and did not lead to non-union, except in the solitary case where the postoperative ROM restriction was not adhered to. Some valuable insights into the surgical technique to reduce this fracture gap have previously been shared by Lee et al. [12], including keeping the transosseous tunnel as thin as possible for smooth suture passage, ensuring that sutures are taut and not crossed prior to tying, positioning knots as close to the patella as possible, incorporating additional non-absorbable suture in a figure-of-eight cerclage pattern or self-tightening suture materials like DYNACORD (DePuy-Synthes Mitek Sports Medicine, Raynham, MA, US). Han et al. recommended a mitigating measure by routinely dissecting the soft tissue between the sutures and bone as far as feasible, followed by cycling the knee to assess if further tightening could be achieved [24].

We did not encounter any instances of implant irritation in either cohort and recommend embedding the suture knots within the quadriceps tendon on the anterior-superior surface of the patella once the knots are secured. Qi et al. proposed that one pole of the drill holes be over-drilled such that the screws act as lag screws to help compress the fracture site, as well as to place the suture in close contact with the bone surface to secure the fixation and avoid suture loosening after soft tissue atrophy [13].

The patient with fibrous non-union was an 84-year-old nursing home resident with poor comorbidities, including osteoporosis undergoing antiresorptive therapy. The potential contributors to non-union in his case were likely fracture displacement from the non-adherence to ROM restrictions and compromised bone quality. Notably, this patient did not undergo any revision surgery, as he could ambulate without pain and had no functional limitations. Within this series, there was no need for implant removal, a trend consistent with or even better than findings in other studies. In the meta-analysis by Huang et al. comparing metallic and non-metallic implants for patellar fractures, 5 patients (6.58%) required additional surgery for non-metallic implant removal, of which 4 cases were for revision due to suture rupture or fracture displacement while 1 case was for implant removal [25].

There was a significant age difference between the all-suture (70.8 ± 10.4 years) and hybrid fixation (54.1 ± 9.6 years) groups. Indeed, hybrid fixation was the obvious choice for older patients who were presumed to have more osteoporotic bone and thus comminuted fractures, necessitating the need for stronger implants in the form of screws to augment the suture fixation of smaller fragments. This not only introduced a selection bias but also highlighted that despite the greater complexity of fracture fixation in the hybrid group, overall outcomes were comparable.

This is the first study to compare all-suture fixation vs. suture fixation with screw augmentation in transverse mid-pole patellar fractures. The surgical technique and postoperative protocol for all patients were also uniform. However, the findings of the study should be interpreted considering its weaknesses too. Given the retrospective nature of this analysis, inherent issues, such as inaccurate or incomplete documentation in medical records and information bias, may have influenced the findings. While we did gather validated functional scores postoperatively, it was prohibitively difficult to retrospectively obtain preoperative scores from patients to show that there was a functional improvement from the surgery. The relatively modest sample size could also have compromised the statistical power of the analysis.

## Conclusions

Hybrid fixation using sutures with screw augmentation for mid-pole transverse patellar fractures proved to be radiographically and functionally comparable to all-suture fixation despite a noticeable age difference between both cohorts, signifying the preference for hybrid fixation for older patients with osteopenic bone. A prospective cohort study with a longer follow-up period would offer a more comprehensive evaluation of outcomes.
